# Ion Dynamics at the Carbon Electrode/Electrolyte Interface: Influence of Carbon Nanotubes Types

**DOI:** 10.3390/ma15051867

**Published:** 2022-03-02

**Authors:** Freddy Escobar-Teran, Hubert Perrot, Ozlem Sel

**Affiliations:** 1 Laboratory Interfaces and Electrochemical Systems, LISE, UMR8235, Sorbonne University, CNRS, F-75005 Paris, France; fescobarteran@hotmail.com (F.E.-T.); ozlem.sel@sorbonne-universite.fr (O.S.); 2 Department of Exact Sciences, The Armed Forces University-ESPE, Sangolqui 171103, Ecuador

**Keywords:** carbon nanotubes, DWCNT, MWCNT, electrochemical quartz crystal microbalance, EQCM, electrode/electrolyte interface, ion transfer

## Abstract

Electrochemical quartz crystal microbalance (EQCM) and AC-electrogravimetry methods were employed to study ion dynamics in carbon nanotube base electrodes in NaCl aqueous electrolyte. Two types of carbon nanotubes, Double Wall Carbon Nanotube (DWCNT) and Multi Wall Carbon Nanotube (MWCNT), were chosen due to their variable morphology of pores and structure properties. The effect of pore morphology/structure on the capacitive charge storage mechanisms demonstrated that DWCNT base electrodes are the best candidates for energy storage applications in terms of current variation and specific surface area. Furthermore, the mass change obtained via EQCM showed that DWCNT films is 1.5 times greater than MWCNT films in the same potential range. In this way, the permselectivity of DWCNT films showed cation exchange preference at cathode potentials while MWCNT films showed anion exchange preference at anode potentials. The relative concentration obtained from AC-electrogravimetry confirm that DWCNT base electrodes are the best candidates for charge storage capacity electrodes, since they can accommodate higher concentration of charged species than MWCNT base electrodes.

## 1. Introduction

The development of energy storage systems (supercapacitors/batteries) to decrease the energy consumption coming from fossil fuels is a way towards a more environmentally friendly society. However, the efficiency of these electrochemical devices depends on the elements constituting them such as the electrode material, which plays an important role to achieve better supercapacitor performances [1,2].

In supercapacitors, the charge storage is based on a reversible adsorption of electrolyte ions towards the surface of electrodes [3,4,5,6,7,8]. Therefore, the selection of the electrode materials is important due to a certain number of parameters such as: specific surface area, porosity, structure, electrical conductivity, surface wettability, and electrochemical stability to improve the performance of electrodes [2,9,10,11,12]. In this way, carbon nanotubes have been used for supercapacitors due to their novel properties such as high electrical conductivity, high charge transport capability, unique pore structure and high specific surface area where the charges are continuously distributed [13,14].

However, the ion dynamics studies at the interfaces are experimentally difficult because there are not many appropriate electrochemical or physico-chemical methods that provide direct access to this kind of information.

Electrochemical quartz crystal microbalance (EQCM) has been extensively used to investigate the charge storage mechanisms in porous materials. EQCM is a powerful in situ technique to measure ionic fluxes at the electrode interfaces, for which current responses (∆*I*) and global gravimetric changes (∆*m*) at the electrode/electrolyte interface are monitored during the electrochemical process. Over the past decade, Levi et al. [15,16,17,18,19,20] have widely employed EQCM to study the charge–compensation mechanism in carbon micropores and, particularly, the effect of specific adsorption of ions with different sizes. Continuing these achievements, EQCM was also used to investigate the charge compensation mechanism between electrode/electrolyte interface [21] and the hydration/solvation effect on the capacitive performance [22,23]. Recently, EQCM has been employed to study the electrolyte concentration effect on the capacitive behavior as well as the compositional changes in porous films [24]. Finally, EQCM combined with nuclear magnetic resonance (NMR) has been employed to understand in a deep manner the charge mechanism in the electrical double layer [25].

Another interesting aspect of the EQCM is the capability to estimate mass and charge variations simultaneously, which provides access to the derivation of the global mass per mole of electrons (MPE) exchanged at the electrochemical interface. In this way, the MPE corresponds to its molar mass when one species is exchanged, but, if multiple ion transfer occurs, EQCM remains limited in interpreting the contribution of different species [22,24]. In order to identify the contribution of different species, EQCM equations were developed incorporating Donnan type electrical double layer models [26]. Furthermore, EQCM with dissipation monitoring (EQCM-D) can be used to study the viscoelastic properties e.g., formation of a solid electrolyte interface (SEI) layer as well as the complex mass changes of the electrodes [27,28,29,30]. This acoustic technique permits to identify the effect of several parameters such as the nature of the electrolytes/ions or the binder on the structure change of the electrodes [31].

Here, an alternative electrochemical and gravimetric method called AC-electrogravimetry was used to complement the EQCM based methods in the energy storage domain. AC-electrogravimetric methodology has been used to study the charge compensation mechanisms in carbon nanotubes [32,33], reduced graphene oxide [34,35], pseudocapacitive metal oxide-based electrodes [25,36] and nanocomposite electrodes [34,37]. Recently, it has been employed to investigate the ion insertion mechanisms in aqueous proton-based batteries [38]. AC-electrogravimetry is a multi-scale coupled electrogravimetric method (quartz crystal microbalance and electrochemical impedance) through which it provides relevant information concerning: (i) identification and kinetic of electroadsorption/desorption of species at the electrode/electrolyte interface, (ii) separation of the charged and non-charged species involved at the electrode/electrolyte interface, and (iii) the relative concentration variations of the species within the material. Therefore, the AC-electrogravimetric methodology was proposed here to study/compare the capacitive behavior in different types of carbon nanotube electrodes. 

## 2. Materials and Methods

### 2.1. Materials

Double Wall CNT (755141-1G, length: 3 μm and diameter: 3.5 nm) and Multi Wall CNT (75517-1G, length: 1 μm and diameter: 9.5) were acquired at the Sigma Aldrich Company (St. Louis, MO, USA).

### 2.2. CNT Thin Films’ Electrode Preparation

CNT films were prepared according to the method described in previous papers [32,34,39]. A solution containing 90% carbon (9 mg) CNT powder and 10% (1 mg) poly(vinylidene fluoride-hexafluoropropylene) (PVDF-HFP) polymer binder in 10 mL of N-methyl-2-pyrrolidone was prepared to elaborate CNT films. Around 8 μL of this solution were deposited through the “drop-casting “method on a gold electrode, which has an effective surface area of 0.20 cm^2^ and remains connected to a quartz crystal resonator (9 MHz-AWS, Valencia, Spain). After that, the carbon films followed a heat treatment with a heating rating of ~5 °C min**^−^**^1^ until 120 °C for 30 min. This treatment was necessary to eliminate the residual solvent and improve the linkage of films on the QCM electrode. The deposited mass was calculated by using the Sauerbrey equation, Δ*f_m_* = −*k_s_*Δ*m*, where Δ*f_m_* is the microbalance frequency change, *k_s_* is the experimental calibration constant (16.3 × 10^+7^ Hz/g·cm^−2^) and Δ*m* corresponds to the mass change. It was obtained by measuring Δ*f_m_*, microbalance frequency change before and after deposition.

### 2.3. Morphological and Physical Characterizations

The CNT powders were characterized by Brunauer–Emmett–Teller methods (BET, Passy, France), X-ray diffraction (XRD, Malvern, United Kingdom) and high-resolution transmission electron microscopy (HR-TEM, Croissy sur Seine, France). Details about the characteristics and selected parameters of these equipment are described in a previous paper [33].

Field emission gun scanning electron microscopy (FEG-SEM, Zeiss, Ultra 55, Jena, Germany) was also employed to investigate the surface morphology of the CNT films. In our experiments, FEG-SEM (Field Emission Gun-Scanning Electron Microscope) provides a very highest resolution imaging compared to regular SEM. The samples were previously prepared on an aluminum stub with a conductive carbon tape and sputter-coated with gold (JEOL JFC-1300 Auto fine coater, Croissy sur Seine, France).

### 2.4. EQCM and AC-Electrogravimetric Characterization

**Figure 1:**
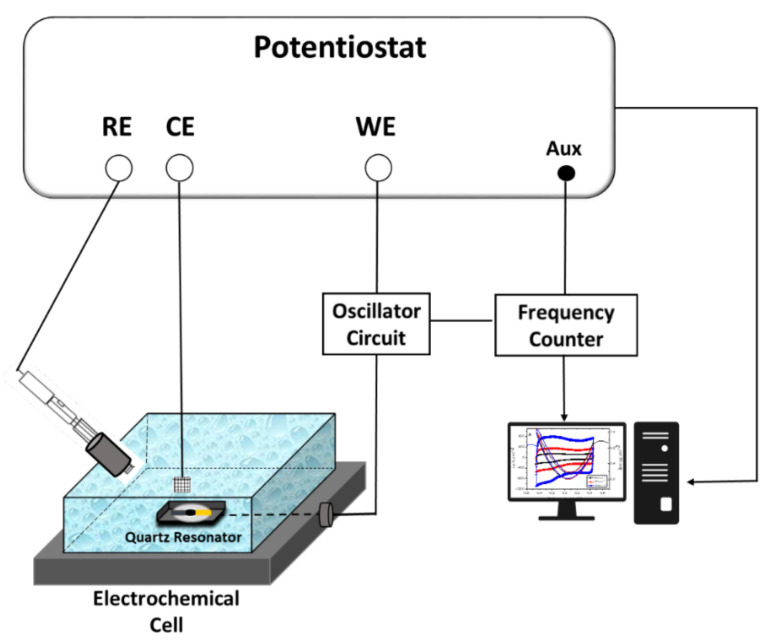
Experimental set-up of an EQCM.

EQCM measurements were carried out in NaCl aqueous solutions and using a three-electrode configuration. A lab-made QCM device (Miller oscillator, Paris, France) was employed to measure frequency shift (∆*f*) of the quartz crystal resonators. A gold electrode of the quartz resonator was used as the working electrode. Platinum grid and Ag/AgCl (3 M KCl) were used as a counter and reference electrode, respectively (See Figure 1). The gravimetric regime was assured by keeping the film thickness acoustically thin (<200 nm).

A QCM set-up connected to a four-channel frequency response analyzer (FRA, Solartron 1254, Élancourt, France) and a lab-made potentiostat (SOLETEM-PGSTAT, Paris, France) were employed to obtain AC-electrogravimetric measurements. The QCM measurements were carried out under dynamic regime, following the potential modulation operating at various frequencies, the electrochemical system being polarized at selected potentials.

In order to obtain a dynamic regime, a sinusoidal small amplitude potential perturbation was superimposed. The frequency intervals were from 63 KHz to 10 mHz. The AC response, ΔI, of the electrochemical system and the mass change, Δ*m*, of the working electrode were simultaneously measured, which resulted in the electrogravimetric TF, *(*∆*m/*∆*E*(*ω*)) and the electrical TF (∆*E/*∆*I(ω)*). These transfer functions were obtained simultaneously at a given potential and frequency modulation, *f* (pulsation, *ω* = 2*πf*). The working principle and AC-electrogravimetric measurement setup have been detailed previously [34,36,40,41].

## 3. Results and Discussion

### 3.1. Material Characteristics

**Figure 2:**
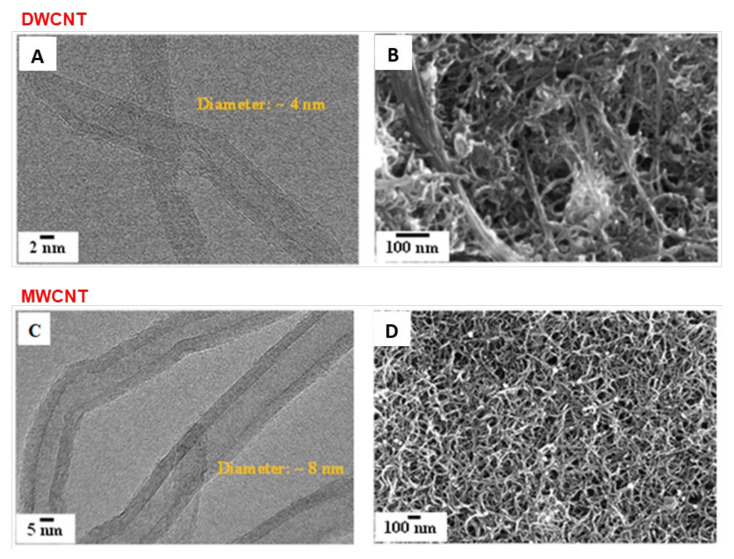
HRTEM images of the DWCNTs (**A**) MWCNTs (**C**) and FEG-SEM images of DWCNT (**B**), MWCNT (**D**) based thin films deposited on the gold patterned quartz resonator.

DWCNTs and MWCNTs constituting the composite electrodes were characterized by HRTEM. The images in Figure 2A,C indicate that the diameters of the DWCNTs and MWCNTs are about ~4 nm and ~8 nm, respectively. Thus, it indicates a large difference between the two samples at this level. Nitrogen sorption measurements and XRD were used to characterize the specific surface area and crystallinity of the CNTs. The Brunauer–Emmett–Teller (BET) specific surface area of the DWCNTs and MWCNTs was estimated to be 552 m^2^·g^−1^ and 300 m^2^ g^−1^, respectively, and the crystallinity attributed to the hexagonal graphitic structure [42] was observed on (002) and (001) reflections (see Figures S1 and S2). BET demonstrates clearly a significant difference between the two materials: DWCNT films give a pore size around 3–4 nm while MWCNTs films show two pore sizes, a first one around 3–4 nm and a second one around 25 nm. On the contrary, the two XRD spectra give similar results, which indicates a similar crystallinity. To assume an equivalent hydrophobic character, successive electrochemical cycles were performed. By this way, an equivalent and optimized electrolyte accessibility is obtained. Then, the CNT electrodes were deposited on the gold patterned quartz resonators. For that, the PVDF-HFP was used as a binder polymer to adhere to the CNT on the gold quartz resonator. Figure 2B,D show an FEG-SEM image of the DWCNT and MWCNT film electrodes, which reveal a high-density of CNT bundles. The surface roughness was not directly estimated, but FEG-SEM images do not show a strong difference about this point between the two films.

### 3.2. EQCM Study of CNTs in 0.5 M NaCl

**Figure 3:**
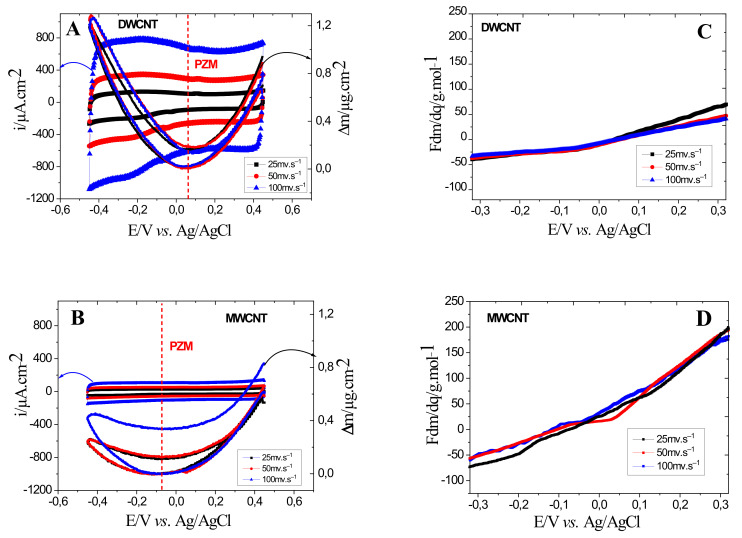
EQCM results of DWCNT (**A**), and MWCNT (**B**) measured in a 0.5 M NaCl aqueous electrolyte. The average molecular weight values of the species involved in the electrochemical process showed as a function of the potential obtained from the reduction branch of EQCM data are presented in (**C**,**D**).

The EQCM results of CNT thin films obtained in aqueous electrolytes of 0.5 M NaCl are shown in Figure 3A, and a growing capacitive current is observed when the scan rate increases (Figure 3A,B) for all the films. In addition, DWCNT films show a higher capacitive current than MWCNT films and even more than SWCNT described in the previous paper [32]. This difference in current is probably due to the pore structure of the materials and its accessibility to the electrolyte ions, which is contradictory with the specific surface area determined on the CNT powder but not with the CNT films. In this study, DWCNT and MWCNT films show quasi-rectangular shaped responses indicating that the charge storage capacity is mainly due to the reversible adsorption/desorption of electrolyte ions. The slight distortion from a perfect rectangular shape is attributed to the presence of a slight faradaic contribution to the charge storage as already mentioned [32,33].

Regarding the mass changes, the DWCNT and MWCNT follow the same order as the current values, i.e., ∆*m_DWCNT_ >* ∆*m_MWCNT_*. In addition, the reversibility of the mass response is better appreciated in DWCNT than in MWCNT (Figure 3A,B), where a large hysteresis was observed in MWCNT. For the DWCNT, the mass change is higher at cathodic potentials than at anodic potentials, while, in MWCNT, the mass change is slightly more significant at anodic potentials than at cathodic potentials. It is also observed that the PZC is shifted slightly towards more cathodic potentials (Figure 3B). The PZC or PZM is the point where the charge/mass is equal to zero, and this corresponds to the potential of 0.075 ± 0.25 V for DWCNT and −0.075 ± 0.25 V for MWCNT follows the following order: PZM_DWCNT_ < PZM_MWCNT_. In other words, DWCNT films show cations exchange preference, while MWCNT films show anions exchange preference in the same potential range. In addition, the V-shape observed in the mass changes (Figure 3A,B) is due to the selective adsorption/desorption of cationic and anionic species in the potential range [32,42].

The average molecular weight values, $\frac{Fdm}{dq}\;\left(=F\frac{dm}{dt}x\frac{1}{i}\right)$, of the species involved in the electrochemical process showed as a function of the potential are obtained from the reduction branch of EQCM data presented in Figure 3A,B.

Figure 3C,D show the average molecular weight values of the electroadsorbed species if only one ion is transferred. The values are calculated as a function of the applied potential in the NaCl electrolyte. The values vary in the range of 50 to −35 g·mol^−1^ and 180 to −70 g·mol^−1^, for the DWCNT and MWCNT thin films, respectively. From the two Figure 3C,D, it should be noted that, for anodic potentials, positive *F*d*m*/d*q* values are estimated, which corresponds to anion contribution. As for cathodic potentials, negative *F*d*m*/d*q* values were calculated indicating the cation contribution. In all the cases, higher or lower atomic weight compared to Na^+^ or Cl^−^ ions are found, which indicates a complex ion transfer behavior associated with solvent contribution. The observed higher values further indicate that the ions are hydrated and/or accompanied by free solvent molecules during their transfer in the same direction. Particularly in MWCNT, higher values at anodic potentials are observed, which could correspond to anions accompanied by free solvent molecules.

### 3.3. AC-Electrogravimetric Study of CNT Thin Film Electrodes in 0.5 M NaCl

**Figure 4:**
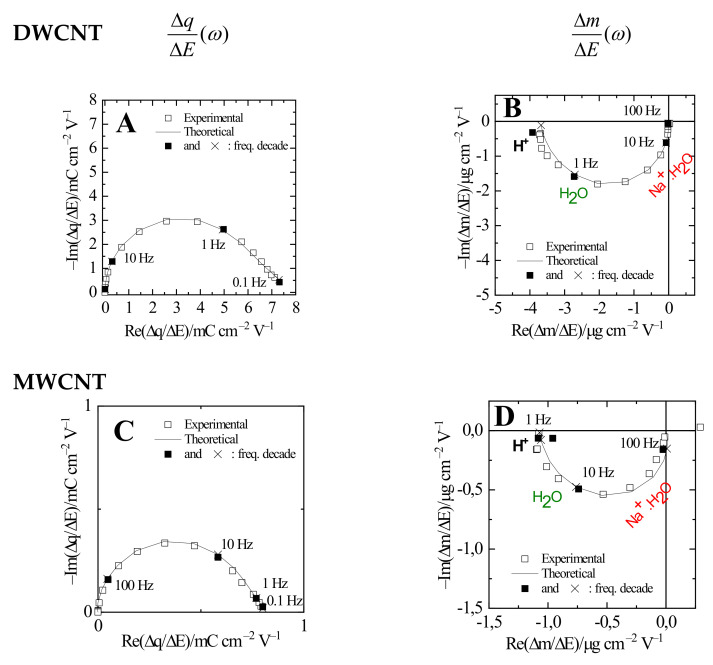
Experimental and theoretical AC-electrogravimetric data of the CNT thin films in 0.5 M NaCl measured at −0.4 V vs. Ag/AgCl. (**A**,**C**) $\frac{\Delta q}{\Delta E}(\omega )$, (**B**,**D**) $\frac{\Delta m}{\Delta E}(\omega )$. The parameter values of *K_i_*, *G_i_* and *Rt_i_* are shown in Table 1.

Analysis of AC-electrogravimetric responses of DWCNT and MWCNT film electrodes are presented in Figure 4 in a comparative manner at a selected potential (−0.4 V) in the cathodic part.

First, the charge/potential transfer functions (TFs), $\frac{\Delta q}{\Delta E}(\omega )$, permit a suitable separation of the ionic contributions, however, without any possibility to identify the ionic species involved.

In Figure 4A and C for DWCNT and MWCNT films, respectively, one big loop is observed, which can be assigned to only one species. However, two species can also be assigned regarding the fact that the time constants of ions are not really different from each other.

To clarify these ideas, the mass /potential transfer functions, $\frac{\Delta m}{\Delta E}(\omega )$, is used. Figure 4B,D show a big loop in the third quadrant from 100 to 1 Hz, which is characteristic of cation and free solvent molecules in the same flux directions, as described previously [40,41].

The experimental TFs in Figure 4 were fitted using Equations (A2) and (A3) (Appendix A): firstly, the number of species intervening and their respective *K_i_* and *G_i_* parameters were estimated from $\frac{\Delta E}{\Delta I}(\omega )$ or $\frac{\Delta q}{\Delta E}(\omega )$. These two key parameters were also used for the fitting of the experimental $\frac{\Delta m}{\Delta E}(\omega )$ TF. Then, identification of the involved species was achieved by fitting the experimental data with the theoretical $\frac{\Delta m}{\Delta E}(\omega )$ TF, which is shown in Equation (A4) where the molar masses of the species (*M_i_*) intervene in this equation. Here, for two films, the molar masse represents H^+^ (c1) and Na^+^.H_2_O (c2) for cations and free solvent (s) molecules in the same flux direction (Figure 4). Furthermore, at selected potential (−0.4 V), there are no anion contributions, which is coherent in this potential region [32,33]. In our experiments, the transport inside the pores is not clearly seen as shown in the EIS responses (Figures S3C and S4C); the typical characteristic slope around 45° slope is not observed here. For this reason, our model does not take into account this effect here.

The presence of two different cationic species and the free solvent molecules contribution estimated by simulating the experimental data was further confirmed by a fair analysis of the partial electrogravimetric transfer functions, for example, by removing the c2 contribution and calculating ${\left.\frac{\Delta q}{\Delta E}\right|}_{th}^{c1s}\left(\omega \right)$, by removing the c1 contribution and calculating ${\left.\frac{\Delta q}{\Delta E}\right|}_{th}^{c2s}\left(\omega \right)$, (Equations (A5) and (A6), no anion contribution at −0.4 V). The fitting results concerning the partial electrogravimetric transfer functions are given in Figures S3 and S4A,B. A good fit also appears for the partial TFs including the same group of parameters, reinforcing the hypothesis of the different ion contributions (Table 1).

In addition, the species contribution was seen previously using electrochemical modulation showing different kinetics of transfer [43] e.g., Hillman et al. monitored the kinetic of species in nickel hydroxide thin films by combining EQCM and probe beam deflection (PBD) [44,45,46].

Figure 4 shows the AC-electrogravimetric results at −0.4 V vs. Ag/AgCl. In fact, it was measured from −0.45 V to 0.45 V vs. Ag/AgCl to have a complete electrochemical exploration of our system. The nature of the species (*M_i_*) and the corresponding *K_i_* and *G_i_* constants were estimated as a function of potential [34,36,47].

**Figure 5:**
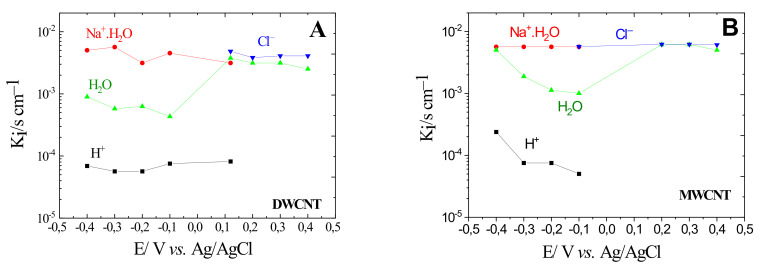
Kinetic constants of interfacial transfer, *K_i_*, DWCNT (**A**), MWCNT (**B**) estimated from the fitting of the AC-electrogravimetric data and measured in aqueous 0.5M NaCl.

Figure 5 shows the variation of the constant transfer kinetics, *K_i_*, of the species as a function of the applied potential. Based on the *K_i_* values presented in Figure 5A,B, the Na^+^.H_2_O and Cl^−^ ions are the fastest of all species for SWCNT and MWCNT films. Here, the number of water molecules associated with the sodium ions is found to be *n* = 1 for all the potentials. Furthermore, the transfer kinetics of free water molecules are somewhat close to the values of the chlorine ions at anode potentials. In addition, these water molecules accompany the transfer of Na^+^.H_2_O and Cl^−^, most likely due to an electrodragging phenomena [32,33].

Finally, the H^+^ ion is the slowest species at cathodic potentials for DWCNT and MWCNT films (Figure 5A,B) showing a similar order of magnitude of *K_i_* values, which is coherent with their substantially lower concentration in the media.

**Figure 6:**
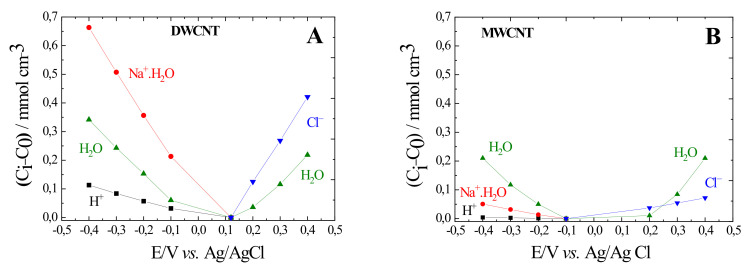
Evolution of the relative concentration, *C_i_–C*_0_, for DWCNT (**A**), MWCNT (**B**) of the charged and non-charged species over the applied potential and measured in aqueous 0.5M NaCl.

In order to quantify the role of each species, ${\left.\frac{\Delta C_{i}}{\Delta E}\right|}_{\omega \to 0}=-\frac{G_{i}}{K_{i}}$ has been calculated as a function of the applied potential using the parameters given by the AC-electrogravimetric fitting. Figure 6 shows the integration of ${\left.\frac{\Delta C_{i}}{\Delta E}\right|}_{\omega \to 0}$ against potential gives the relative concentration change, *(C_i_–C*_0_*)* of the charged and non-charged species. For MWCNTs (Figure 6B), the *(C_i_–C*_0_*)* values of the H_2_O are higher than the *(C_i_–C*_0_*)* values of the Na^+^.H_2_O, Cl^−^ and H^+^.

In contrast to MWCNT, in DWCNTs, the evolution of relative concentration of species presents a different trend. In comparison with the other species, the *(C_i_–C*_0_*)* values of the Na^+^.H_2_O are higher than the *(C_i_–C*_0_*)* values of H^+^ and H_2_O at cathode potentials (Figure 6A). In conclusion, these results indicate that DWCNTs appear to be the best candidate for charge storage capacity electrodes, since it can accommodate a higher concentration of charged species in DWCNTS than in MWCNTs. This result can be attributed to the difference of material form illustrated by the difference of specific surface, which is higher in the case of the DWCNTs, almost two times higher, compared with the value of the MWCNTs. A higher capacitance was also found, in comparison with values given by compact films, with different nanostructured materials as titanium dioxide based composite nanotube-arrays, MnO_2_ electrodes, or activated carbon nanoparticles [48,49,50].

## 4. Conclusions

Carbon nanotubes (CNTs) of “double-walled” (DWCNT), and “multi-walled” (MWCNT) films were elaborated on gold electrodes of microbalance and tested in NaCl aqueous electrolyte. The effect of pore morphology/structure but also roughness or hydrophilicity over the classical electrochemical responses demonstrated that DWCNT are better candidates for energy storage applications than MWCNT base electrodes in terms of current/mass variation and permselectivity of cations. The mass change obtained via EQCM showed that DWCNT films are 1.5 times bigger than MWCNT films in the same range of potential. In this way, the permselectivity of DWCNT films showed cation exchange preference for cathode potentials while MWCNT films showed anion exchange preference for anode potentials. The relative concentration obtained from AC-electrogravimetry confirms that DWCNT base electrodes are the best candidates for polarizable electrodes, since they can accommodate a higher concentration of charged species than MWCNT and even more than SWCNT base electrodes described in the previous paper [32].

## Figures and Tables

**Table 1:** *K_i_* (kinetics of transfer), and *G_i_* (inverse of the transfer resistance), *Rt_i_* (transfer resistance) values extracted from the fitting results from AC-electrogravimetric measurements in aqueous 0.5 M NaCl at –0.4 V vs. Ag/AgCl for DWCNT and MWCNT films.

CNT Types/ Species	*M_i_* (g·mol^−1^)	Species Identification	*K_i_* (cm·s^−1^)	*G_i_* (mol· s^−1^·cm^−2^·V^−1^)	*Rt_i_ = 1/FG_i_* ohm·cm^2^
**DWCNT**					
*c*2	23 + 18	Na^+^.H_2_O	2.89 × 10^−3^	4.65 × 10^−6^	2.22
*s*	18	H_2_O	6.28 × 10^−4^	9.11 × 10^−7^	11.37
*c*1	1	H^+^	3.64 × 10^−5^	1.39 × 10^−8^	745.52
**MWCNT**					
*c*2	23 + 18	Na^+^.H_2_O	5.66 × 10^−3^	1.10 × 10^−6^	9.42
*s*	18	H_2_O	5.03 × 10^−4^	5.23 × 10^−6^	1.98
*c*1	1	H^+^	2.39 × 10^−4^	3.58 × 10^−9^	2894.61

## Supplementary Materials

The following supporting information can be downloaded at: https://www.mdpi.com/article/10.3390/ma15051867/s1. Figures S1 and S2 XRD Spectra, Nitrogen sorption isotherm and the pore size distribution (PSD). Figures S3 and S4 experimental and theoretical AC-electrogravimetric data of the CNT thin films.

## Appendix A

For different types of CNT films, the concentration transfer function, ${\left.\frac{\Delta C_{i}}{\Delta E}\right|}_{th}\left(\omega \right)$, of cation1 (*c*1), cation 2 (*c*2), anion (*a*), and free solvent (*s*)) were calculated through:

(A1) $${\left.\frac{\Delta C_{i}}{\Delta E}\right|}_{th}\left(\omega \right)=\frac{-G_{i}}{j\omega d_{f}+K_{i}}$$

where *ω* = 2π*f* is the pulsation, *d_f_* is the film thickness, *K_i_* represents the kinetics transfer, whereas *G_i_* describes the ease/difficulty of ionic species (*c*1 and *c*2) to be transferred at the electrode/electrolyte interface. In addition, the transfer resistance, *Rt_i_,* can be determined through *G_i_* using the following expression: *Rt_i_* = 1/*FG_i_*, where *F* is the Faraday number [38,39,40].

The experimental data of the electrochemical impedance ${\left.\frac{\Delta E}{\Delta I}\right|}_{th}\left(\omega \right)$ and the charge/potential TF, ${\left.\frac{\Delta q}{\Delta E}\right|}_{th}\left(\omega \right)$, were fitted using theoretical transfer functions given in Equations (A2) and (A3). In this case, two cations, *c*1 and *c*2 and anion, *a*, are involved in the charge compensation process:

(A2) $${\left.\frac{\Delta E}{\Delta I}\right|}_{th}\;\left(\omega \right)={\left[j\omega d_{f}\left(\frac{G_{c1}}{j\omega d_{f}+K_{c1}}+\frac{G_{c2}}{j\omega d_{f}+K_{c2}}-\frac{G_{a}}{j\omega d_{f}+K_{a}}\right)\right]}^{-1}$$

(A3) $${\left.\frac{\Delta q}{\Delta E}\right|}_{th}\;\left(\omega \right)=d_{f}F\left(\frac{G_{c1}}{j\omega d_{f}+K_{c1}}+\frac{G_{c2}}{j\omega d_{f}+K_{c2}}-\frac{G_{a}}{j\omega d_{f}+K_{a}}\right)$$

The theoretical electrogravimetric transfer function, ${\left.\frac{\Delta m}{\Delta E}\right|}_{th}\left(\omega \right)$, can be estimated, taking into account the charged/uncharged species contribution:

(A4) $${\left.\frac{\Delta m}{\Delta E}\right|}_{th}\;\left(\omega \right)=d_{f}\left(M_{c1}\frac{G_{c1}}{j\omega d_{f}+K_{c1}}+M_{c2}\frac{G_{c2}}{j\omega d_{f}+K_{c2}}+M_{c2}\frac{G_{a}}{j\omega d_{f}+K_{a}}+M_{s}\frac{G_{s}}{j\omega d_{f}+K_{s}}\right)$$

In Equation (A4), *M_c_*_1_*, M_c_*_2_*, M_a_* and *M_s_* are the atomic weight of involved species.

From the theoretical overall electrogravimetric transfer function (Equation (A4)), it is possible to calculate the theoretical partial transfer functions by removing the *c*2 contribution, calculating ${\left.\frac{\Delta m}{\Delta E}\right|}_{th}^{c1as}\;\left(\omega \right)$; or the *c*1 contribution, calculating; ${\left.\frac{\Delta m}{\Delta E}\right|}_{th}^{c2as}\;\left(\omega \right)$; or the anion contribution calculating; ${\left.\frac{\Delta m}{\Delta E}\right|}_{th}^{c1c2s}\;\left(\omega \right)$, as shown in the following equations:

(A5) $${\left.\frac{\Delta m}{\Delta E}\right|}_{th}^{c1as}\;\left(\omega \right)=d_{f}\left((M_{c1}-M_{c2})\frac{G_{c1}}{j\omega d_{f}+K_{c1}}+(M_{a}-M_{c2})\frac{G_{a}}{j\omega d_{f}+K_{a}}+M_{s}\frac{G_{s}}{j\omega d_{f}+K_{s}}\right)$$

(A6) $${\left.\frac{\Delta m}{\Delta E}\right|}_{th}^{c2as}\;\left(\omega \right)=d_{f}\left((M_{c2}-M_{c1})\frac{G_{c2}}{j\omega d_{f}+K_{c2}}+(M_{a}-M_{c1})\frac{G_{a}}{j\omega d_{f}+K_{a}}+M_{s}\frac{G_{s}}{j\omega d_{f}+K_{s}}\right)$$

(A7) $${\left.\frac{\Delta m}{\Delta E}\right|}_{th}^{c1c2s}\;\left(\omega \right)=d_{f}\left((M_{c1}-M_{c2})\frac{G_{c1}}{j\omega d_{f}+K_{c1}}+(M_{c2}-M_{c1})\frac{G_{c2}}{j\omega d_{f}+K_{c2}}+M_{s}\frac{G_{s}}{j\omega d_{f}+K_{s}}\right)$$

These last estimated partial transfer functions correspond to a key point of our AC-electrogravimetric technique. Indeed, this cross-checking allows a fair validation of our measurements to be performed.
