# Supplementary material for: Ion Dynamics at the Carbon Electrode/Electrolyte Interface: Influence of Carbon Nanotubes Types
Source: Materials (Basel). 2022 Mar 2;15(5):1867. doi: 10.3390/ma15051867 (PMC8912032; doi:10.3390/ma15051867)
Supplement: Supplementary file 1 [file materials-15-01867-s001.zip › materials-1526641-supplementary.pdf]

materials

Supporting Information

Ion Dynamics at the Carbon Electrode/Electrolyte Interface: Influence of Carbon Nanotubes Types.

Freddy Escobar-Teran [1,2], Hubert Perrot [1,*] and Ozlem Sel [1]

[1] Laboratory Interfaces and Electrochemical Systems,LISE, UMR8235, Sorbonne University, CNRS, F-75005 Paris, France; fescobarteran@hotmail.com (F.E.T.); ozlem.sel@sorbonne-universite.fr (O.S.)
[2] Department of Exact Sciences, the Armed Forces University -ESPE, 171103 Sangolqui, Ecuador
* Correspondence: hubert.perrot@sorbonne-universite.fr

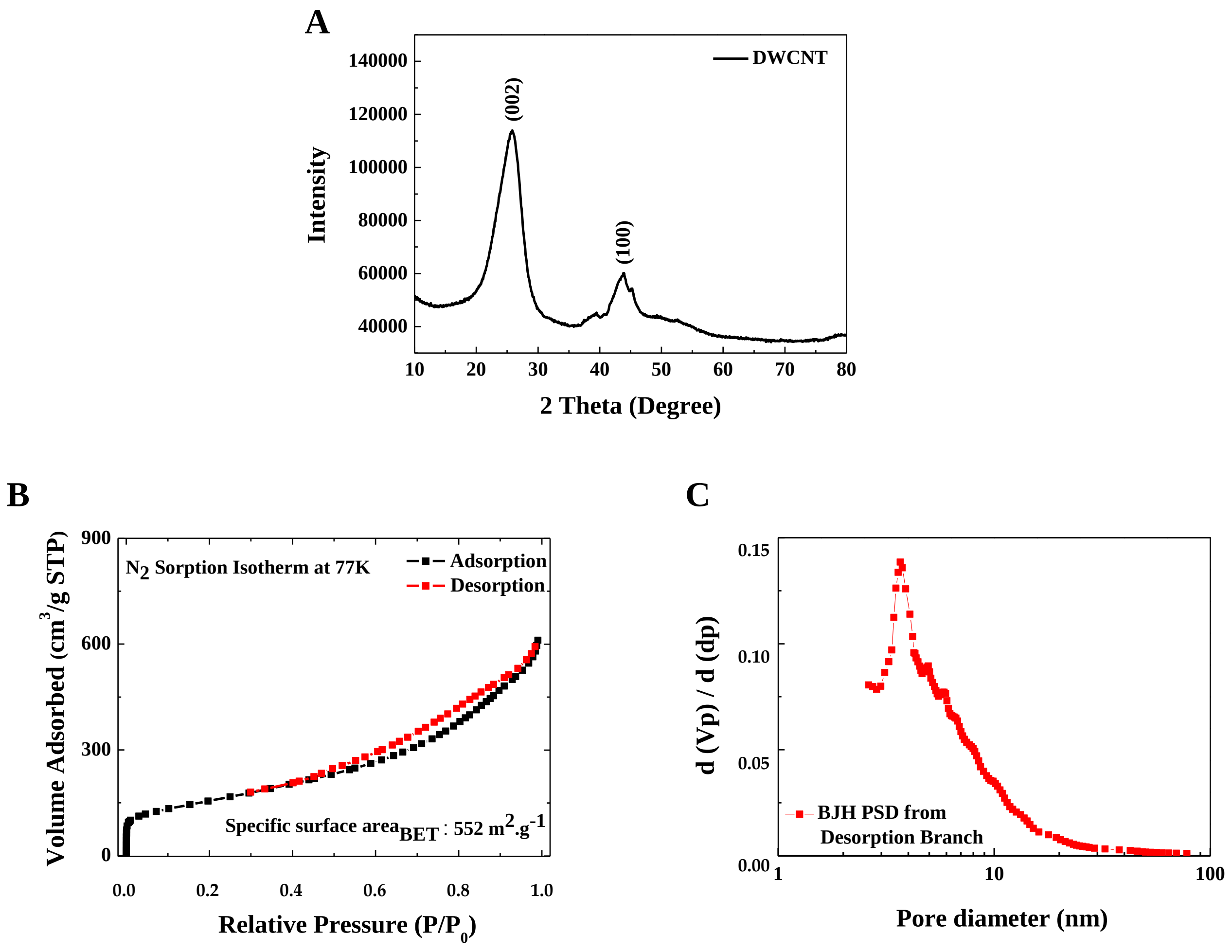

Figure S1. XRD spectra (**A**) of DWCNTs. Nitrogen sorption isotherm (**B**) and the pore size distribution (PSD) (**C**) of DWCNTs.

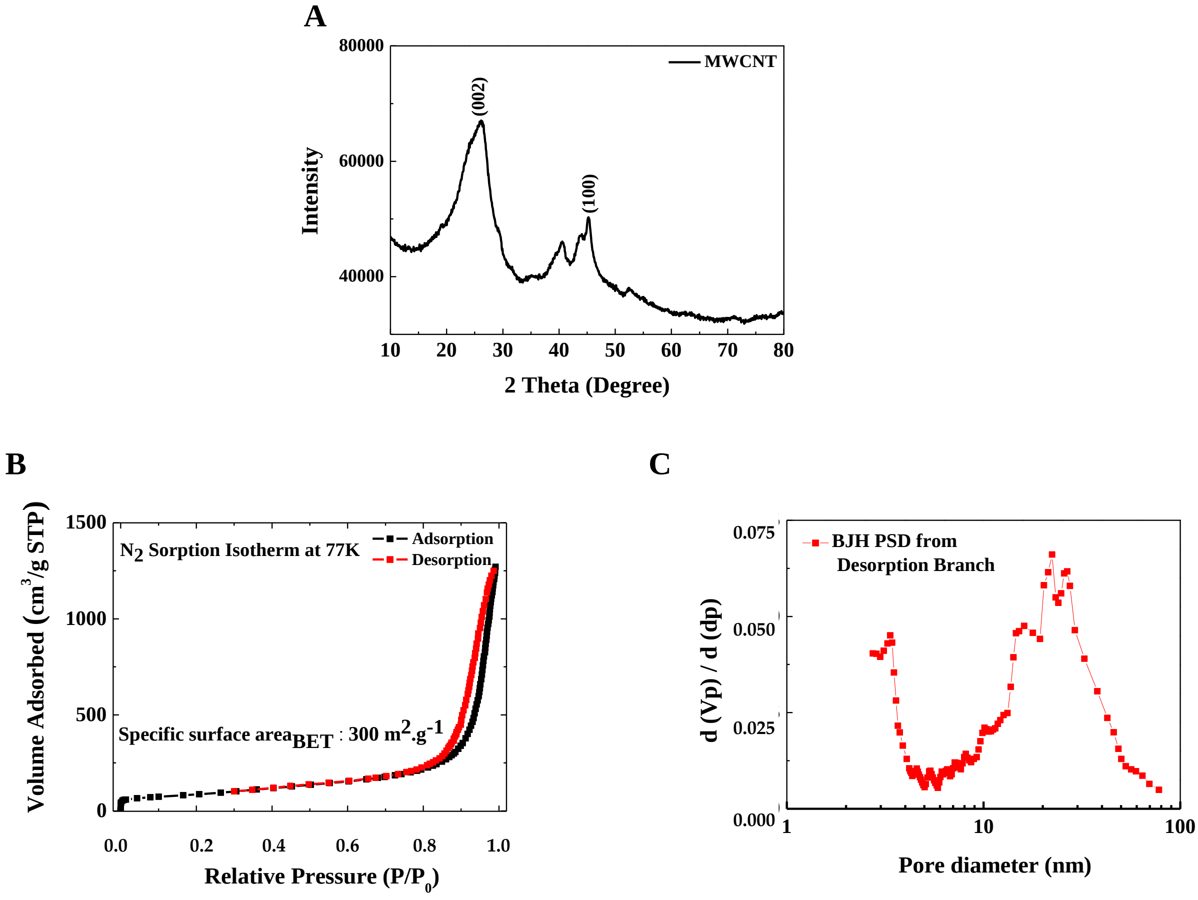

Figure S2. XRD spectra (**A**) of MWCNTs. Nitrogen sorption isotherm (**B**) and the pore size distribution (PSD) (**C**) of MWCNTs.

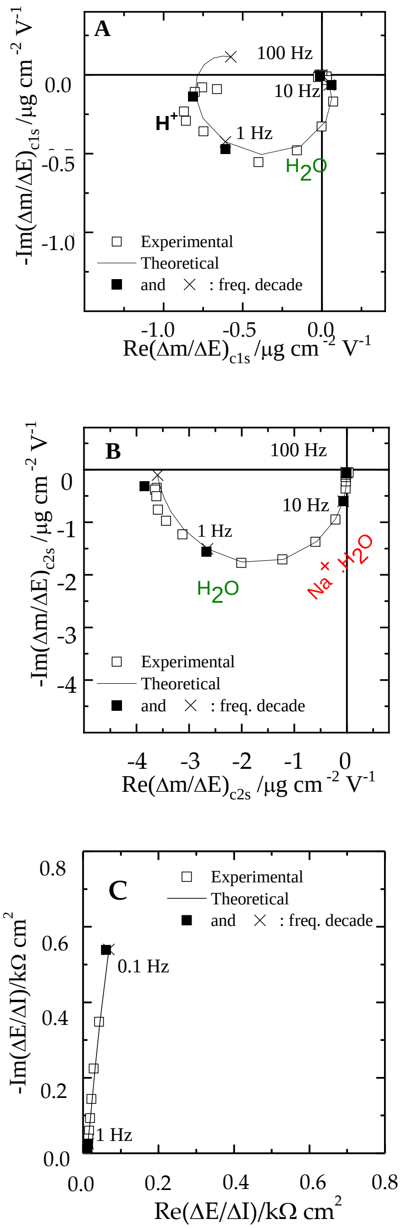

Figure S3. Experimental and theoretical AC-electrogravimetric data of the DWCNT thin film in 0.5 M NaCl measured at −0.4 V vs. Ag/AgCl. (**A**) $\frac{\Delta m}{\Delta E}\Big|^{c1s}(\omega)$, (**B**) $\frac{\Delta m}{\Delta E}\Big|^{c2s}(\omega)$, (**C**) $\frac{\Delta E}{\Delta I}(\omega)$. Theoretical functions were calculated with the following parameters: d_f= 0.4µm, K_{c1} = 3.64 x 10^{-5} cm.s^{-1}, G_{c1} = 1.39 x 10^{-8} mol.s^{-1}.cm^{-2}.V−1, K_{c2} = 2.89 x 10^{-3} cm.s^{-1}, G_{c2} = 4.65 x 10^{-6} mol.s^{-1}.cm^{-2} V^{-1}, K_s = 6.28 x 10^{-4} cm.s^{-1}, G_s = 9.11 x 10^{-7} mol.s^{-1}, m_{c1} = 1 g.mol^{-1}, m_{c2} = 23+18 g.mol^{-1} and m_s = 18 g.mol^{-1}.

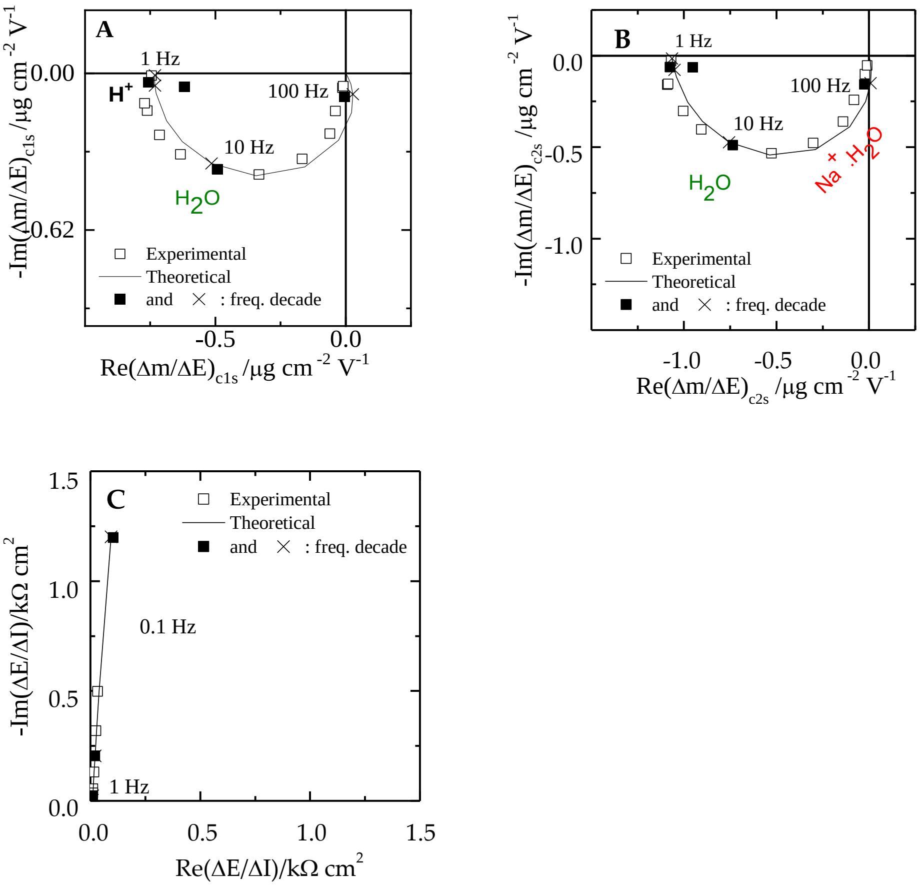

Figure S4. Experimental and theoretical AC-electrogravimetric data of the MWCNT thin film in 0.5 M NaCl measured at −0.4 V vs. Ag/AgCl. (**A**) $\frac{\Delta m}{\Delta E}\Big|^{c1s}(\omega)$, (**B**) $\frac{\Delta m}{\Delta E}\Big|^{c2s}(\omega)$, (**C**) $\frac{\Delta E}{\Delta I}(\omega)$. Theoretical functions were calculated with the following parameters: d_f= 0.4µm, K_{c1} = 2.39 x 10^{-4} cm.s^{-1}, G_{c1} = 3.58 x 10^{-9} mol.s^{-1}.cm^{-2} V^{-1}, K_{c2} = 5.66 x 10^{-3} cm.s^{-1}, G_{c2} = 1.10 x 10^{-6} mol.s^{-1}.cm^{-2}.V^{-1}, K_s = 5.03 x 10^{-4} cm.s^{-1}, G_s = 5.23 x 10^{-6} mol.s^{-1}, m_{c1} = 1 g.mol^{-1}, m_{c2} = 23+18 g.mol^{-1} and m_s = 18 g.mol^{-1}.
